# Histone Butyrylation/ Acetylation Remains Unchanged in Triple Negative Breast Cancer Cells after a Long Term Metabolic Reprogramming

**DOI:** 10.31557/APJCP.2019.20.12.3597

**Published:** 2019

**Authors:** Fatemeh Mehdikhani, Hossein Ghahremani, Saeedeh Nabati, Haniyeh Tahmori, Majid Sirati-Sabet, Siamak Salami

**Affiliations:** *Department of Clinical Biochemistry, School of Medicine, Shahid Beheshti University of Medical Science, Tehran, Iran. *

**Keywords:** Triple negative breast cancer, cellular metabolism, histone butyrylation, beta-hydroxybutyrate

## Abstract

**Background:**

Altered metabolism is one of the hallmarks of the cancer cells which reciprocally interrelate with epigenetic processes, such as post-translational histone modifications to maintain their desired gene expression profiles. The role of beta-hydroxybutyrate as a ketone body in cancer cell biology and histone modifications are reported. The present study aimed to evaluate the impacts of long-term metabolic reprogramming via glucose restriction and beta-hydroxybutyrate treatment on histone acetylation and butyrylation in MDA-MB231 cells as a model of triple negative stem-like breast cancer.

**Methods:**

For long-term treatment, cells were set up in three groups receiving DMEM with restricted glucose (250 mg/L), DMEM with restricted glucose but enriched with five millimolar beta-hydroxybutyrate and DMEM with standard glucose (1g\L) and investigated for a month. Histone modifications, including H3 acetylation and butyrylation, were investigated by immunoblotting after an acid extraction of the histone proteins.

**Results and Conclusion:**

Neither beta-hydroxybutyrate enrichment nor glucose restriction elicited a significant effect on the butyrylation or acetylation level of histone H3 upon a long-term treatment. Metabolic plasticity of cancer cells, mainly stem-like triple negative breast cancer cells alleviate or neutralize the impact of long-term metabolic reprogramming via restriction of glucose and histone modifications enrichment. These results shed new light upon the mechanism of controversial efficacy of ketogenic diets in clinical trials.

## Introduction

Cancer is still one of the leading causes of death worldwide, and breast cancer is one of the most common types of cancer in the well-developed countries, but over the past 30 years, the trend of breast cancer deaths in developing countries has been increasing (Torre et al., 2016). Breast cancer, based on the expression of estrogen and progesterone receptors, and HER-2, are divided into subgroups in which the subgroup lacking those markers are called triple negative breast cancer (TNBC). This type of breast cancer accounts for 10% to 20% of all types of breast cancer, and at the time of diagnosis, they have a high mitotic index, poor prognosis and less response to the conventional treatments (Salami et al., 2011; Engebraaten et al., 2013; Kumar and Aggarwal, 2016). TNBCs are part of basal-like breast cancers which are less differentiated and look a lot like a small population of cancer cells known as the cancer stem cells (CSCs) (Ihemelandu et al., 2007).

In contrast to other cancer cell populations, CSCs have specific characteristics that make them more aggressive and more resistant to the current therapies. Such therapies mainly eliminate the dominant sensitive populations of cancer cells which provide better access to the metabolic fuels for remaining resistant CSC and prevail in cancer recurrence and metastases(Farrar, 2010; Velasco-Velázquez et al., 2011). Among solid tumors, CSCs was firstly identified in breast cancer as the cells with CD44^+^CD24^low-^ phenotype (Al-Hajj et al., 2003). 

It is well documented that the formation of a cancer cell is accompanied by an increase in the accumulation of genetic and epigenetic changes which gradually confer strong proliferative, and aggressive properties (You and Jones, 2012). Epigenetic changes such as CpG island DNA methylation and post-translational changes of histone proteins are related to changes in cell phenotype without alteration in the DNA sequence that alters genes expression (Bártová et al., 2008). Post-translational changes such as histone acylation (such as acetylation, methylation, propionylation, butyrylation, crotonylation), have occurred in histone proteins and cause silencing or activation of the gene expressions (Mai et al., 2005). Inappropriate regulation of DNA and post-translational modifications of histones are the frequent occurrences in a large number of diseases, including cancer and neurological abnormalities (Dawson and Kouzarides, 2012). Butyrylation has been recently studied in different physiological conditions, such as the differentiation of germ cells and revealed that, like acetylation, it could enhance the expression of certain genes (Goudarzi et al., 2016). Recent studies showed a solid reciprocating interrelationship between metabolism and post-translational modification of histone proteins (Simithy et al., 2017).

For years, we know that cancer cells convert glucose to lactate in the presence or absence of oxygen, a phenomenon known as the Warburg effect, which produces ATP via glycolysis. Increasing the conversion of pyruvate to lactate during aerobic glycolysis results in acidosis in the tumor microenvironments that facilitates invasion and metastasis of cancer cells (Shukla et al., 2014; Salamon et al., 2017; San-Millan and Brooks, 2017). However, metabolic plasticity is a newfound hallmark of cancer cells which shows that how different population of cancer cells within a tumor are using different metabolic pathways. Elucidation of characteristics of cancer cell metabolism and tailoring the therapeutic strategies are part of current scientific efforts (Peiris-Pagès et al., 2016; Danhier et al., 2017; Schwartz et al., 2017; Riester et al., 2018). 

In addition to glucose, ketone bodies, in the process of intrinsic ketosis due to starvation and prolonged exercise, work as alternative fuels (Evans et al., 2017). However, extrinsic ketosis by using ketogenic diet can also increase serum beta-hydroxybutyrate levels (van Delft et al., 2010). Ketogenic diet by restricting calories from the source of carbohydrates and providing a source of fat leads to induction of ketosis in the recipient (Freeman et al., 1998). Clinical trials and experimental studies have shown that beta-hydroxybutyrate has anti-cancer and anti-inflammatory effects (Ruskin et al., 2009; Allen et al., 2014; Woolf et al., 2016). Studies have shown that dietary ketones in metastatic cancer mice increase survival rates by 70-60% (Poff et al., 2014).

On the other hand, it has shown that the use of ketones could lead to tumor growth and metastasis (Martinez-Outschoorn et al., 2012b). Contradictory results reveal that the precise mechanism of the function of this type of chemicals in cancer biology is still not well defined. Evidence of the interrelationship between ketone bodies and the regulation of epigenetic modifications has also been reported, which can provide a mechanism for linking energy metabolism in cells and regulating gene expression through histone modifications (Sabari et al., 2017; Li et al., 2018).

The current challenges in the treatment of stem-like triple negative breast cancers and the ambiguity in the impacts of beta-hydroxybutyrate on the post-translational modification of histones, motivated us to investigate the effect of beta-hydroxybutyrate as a ketone body on the occurrence of histone butyrylation of MDA-MB231cells as a model of stem-like triple negative breast cancer.

## Materials and Methods 

In this research, the MDA-MB231 cell line was purchased from the Iranian Banking Bank (NCBI) as the TNBC cell line with the CD44highCD24^low / -^ phenotype. The authentication of the cell line was certified by NCBI via STR studies. Initially, cells were cultured in low glucose DMEM medium and were incubated in a CO_2_ incubator at 37°C, 96% humidity and 5% CO_2_. Culture vessels were regularly examined for growth and contamination.

*Cytotoxicity study*

The MTT assay was used to evaluate cytotoxicity. For an MTT assay, 5000 cells were seeded in each well of a 96-well plate. The plate was placed in an incubator for 24 hours to allow the cells to attach to the wells. Cells were treated with a range of beta-hydroxybutyrate (Sigma Aldrich LLC, MW = 126) concentrations between 25 and 1,000 mM in PBS at 37 ° C for 48 hours. MTT solution was added in each well, and the plate was incubated for 4 hours. The formazan crystals were dissolved entirely by adding DMSO. The absorbance was read at 595 nm and 620 nm reference wavelengths using the multimodal reader. The value of IC_50_ was obtained by non-linear fitting using GraphPad Prism 6 software.

*Cell treatment*

For a long-term study, cells were set up in controls (DMEM medium with 1 g/L glucose), ¼ glucose-restricted cells (DMEM with 250 mg/L glucose), and ¼ glucose-restricted cells enriched with 5 mM beta-hydroxybutyrate and all were maintained for 30 days. 

In all studies, culture media were enriched with 10% FBS, glutamine and bicarbonates. 

*Extraction of histones*

Cells were Scraped and washed in PBS. Then, they were lysed using RIPA lysis buffer containing appropriate ratio of protease inhibitor cocktail (X100) and were sonicated twice for 5 seconds (power 50% and duty 50%) on ice. The supernatant containing the total protein was extracted by final centrifugation. Sulfuric acid (0.2 M) was added to the total protein and samples were incubated in 4°C overnight on a roller mixer. The next day, the samples were centrifuged (20,000 g) for 15 minutes at and 4°C. The supernatant was transferred to new tubes and the histone proteins were precipitated by TCA (20 %.) after 30 minutes incubation on ice and a 10 minutes centrifugation (x4,000g) at 4°C. The precipitates were washed with 0.1% acetone / HCl and pure acetone. Finally, the precipitates were solved in urea (8 M). The extracted proteins were quantified using Bradford reagent and Bovine serum albumin (BSA) as the standard.

*Protein electrophoresis*

For SDS-PAGE, a 15% separation and a 5% stacking gel were cast and run using Tris-glycine buffers. Five to ten microgram of total protein and four to eight microgram of acid extracted histones were loaded, and the electrophoresis was first performed for 30 minutes at 80 volts and then for 45 minutes at 120 volts. Then, the gel was stained for 1 hour with Coomassie Blue R-250 and stripped off using SDS-PAGE destaining solution (Methanol 30%-Acetic Acid 10%). 

*Immunoblotting*

Butyrylation and acetylation of histones were assessed by using immunoblotting or western blotting. The proteins were transferred to nitrocellulose membranes using a wet transfer tank at a voltage of 90 V for an hour. Ponceau S staining was performed to ensure that the proteins were transferred to the membrane and stripped off using TBS-T buffer. The membranes were blocked using 5% Skimmed Milk or 5% BSA. Pan anti-butyryllysine (K-Bu, PTM-329), Pan anti-acetyllysine (K-Ac, PTM-105), Histone H3 (PTM-1001) and H4 antibodies (PTM-1003, PTM Biolabs) were used as the primary antibodies with 1:1,000 dilution in 5% Skimmed Milk or 5% BSA. Appropriate HRP-conjugated secondary antibodies were utilized to detect the primary antibodies, and the bands were visualized by ECL (GE Healthcare) solution on X-ray films. 

## Results

**Figure 1:**
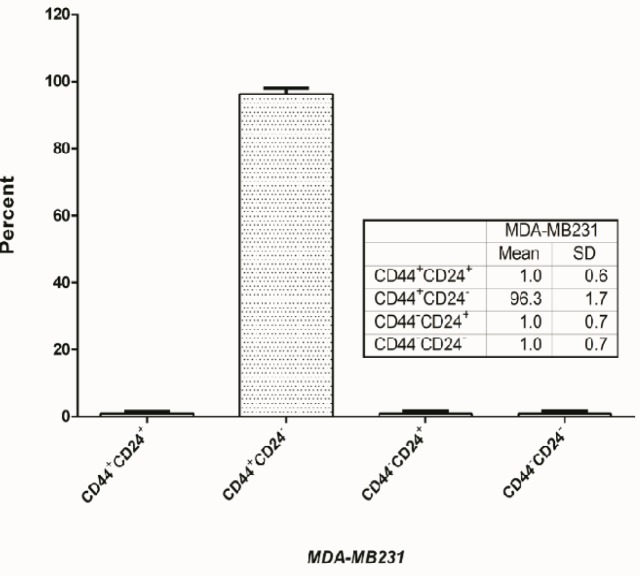
Status of Breast Cancer Stem Cell Markers, CD44 and CD24, in MDA-MB231 Cells. Result showed that more than of 96.3±1.7% cells are cells with CD44+CD24^-/low^ phenotype

**Figure 2:**
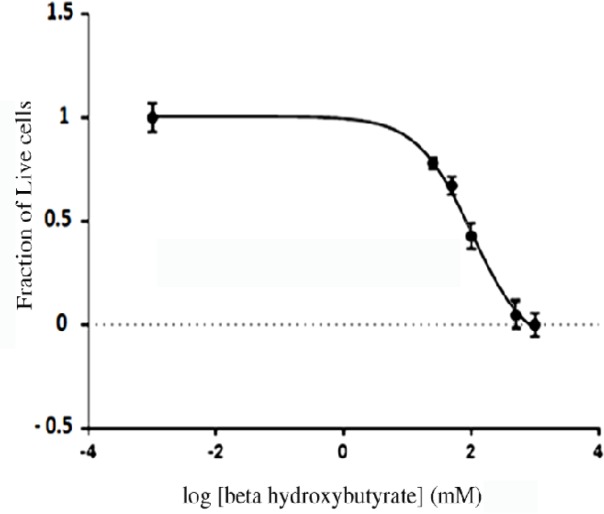
Cell Toxicity of Beta-Hydroxybutyrate. The cellular toxicity of bHB was assessed by MTT assay and revealed an IC_50_ value of 103.1 mM

Glucose is the primary source of energy in cancer cells (Salamon et al., 2017). Ketone bodies are the alternative fuels that could be used as a source of energy during starvation or strenuous exercises. However, studies showed that ketogenic diets elicit beneficiary impacts on patients with different diseases including cancers. Beta-hydroxybutyrate is the most abundant and stable intrinsic ketone body, and the number of its salts offered on the over the counter supplements’ market is steadily increasing (Newman and Verdin, 2014). Hence, the goal of therapeutic ketogenic diets is to achieve a concentration of 3 to 6 mM beta-hydroxybutyrate in an induction period of 7 days and could be sustained for one to three months. The impact of beta-hydroxybutyrate on epigenetic changes such as post-translational histone modifications and gene expressions have been reported (Newman and Verdin, 2017). Histone butyrylation is also a significant histone modification that may regulate the expression of specific genes. Goudarzi (2016) showed that butyrylation, like acetylation, leads to increased gene expression of reproductive cells in male rats. So, in this study the status of CD24-CD44 breast cancer stem cell markers was studied in MDA-MB231 triple negative breast cancers (Figure 1) and due to absolute dominance of CD44^+^CD24^low/-^ phenotype (96.3%±1.7), the cell line was used without further cell sorting. The impact of long-term treatment (one month) of a triple negative stem-like breast cancer cells with a 5 M concentration of beta-hydroxybutyrate. The toxicity of beta-hydroxybutyrate was measured by MTT assay, and its IC_50_ value was 103.1 mM (Figure 2). No significant toxicity was observed in doses less than 20 mM.

**Figure 3:**
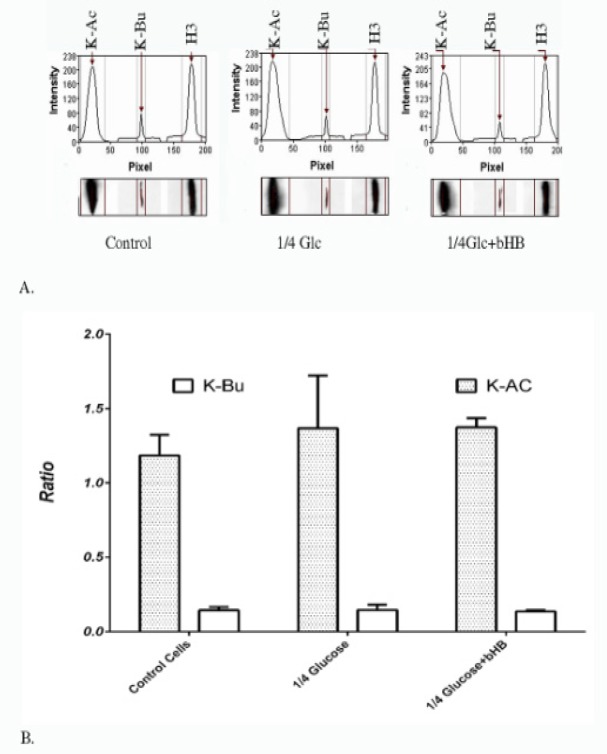
Immunoblotting of Post - Translational Modification of Histones after Long-Term Treatment. A. Densitometric analyses were done using 1D gel electrophoresis image analysis software GelAnalyzer. B. No significant changes were observed in K-Ac or K-Bu of histone H3 in ¼ glucose-restricted cells (DMEM with 250 mg/L glucose) and ¼ glucose-restricted cells enriched with 5 mM beta-hydroxybutyrate. Significant differences was also not observed between ¼ glucose-restricted cells (DMEM with 250 mg/L glucose) and ¼ glucose-restricted cells enriched with 5 mM beta-hydroxybutyrate

## Discussion

After a long-term treatment, the assessment of K-Ac and K-Bu in Histone H3 in MDA-MB231 cells was not revealed any significant changes in cells with restricted access to glucose or even after enrichment with 5 M bHB (Figure 3a). In comparison to controls, significant changes were not observed in K-Ac or K-Bu of histone H3 in ¼ glucose-restricted cells (DMEM with 250 mg/L glucose) ( p=0.2806 and p=0.9998, respectively) and ¼ glucose-restricted cells enriched with 5 mM beta-hydroxybutyrate (p=0.2604 and p=0.9972, respectively) (Figure 3b). No significant differences were also observed between ¼ glucose-restricted cells (DMEM with 250 mg/L glucose) and ¼ glucose-restricted cells enriched with 5 mM beta-hydroxybutyrate (p=0. 0.9983, p= 0.9957, respectively). In the other study, it has been shown that starvation for 48 hours in C57BL6 mice increased blood beta-hydroxybutyrate, thereby increased the amount of beta-hydroxybutyrylation of histone and the expression of the hunger responses genes. They also showed that histone beta-hydroxybutyrylation was increased dose-dependently in HEK293 cells after treatment with beta-hydroxybutyrate(Shimazu et al., 2013). However, they did not study the butyrylation in mice or HEK293 cells.

In the other study using MCF-7 cells, it revealed that bHB increases the amount of histone acetylation which in turn increases transcription of genes (Martinez-Outschoorn et al., 2012a). The HDAC inhibitory effect of bHB was also could lead to increased histone acetylation (Shimazu et al., 2013). On the other hand, it has also shown that there is no change in the amount of histone acetylation with increasing beta-hydroxybutyrate levels, and only in high concentrations of beta-hydroxybutyrate induces a slight change in the amount of histone acetylation (Xie et al., 2016). Our findings with MDA-MB231 cells is consistent with the results of the later study. Such inconsistency in the results could be aroused from the different composition capacity of ketone bodies in different cells and tissue (Ruan and Crawford, 2018). 

It is proved that differential histone acylation is regulated by metabolism of the different acyl-CoA forms, which in turn modulate the regulation of gene expression and crotonyl can work as an intracellular source for beta-hydroxybutyrate under normal conditions within the cells by CDYL enzyme (Liu et al., 2017). This finding indicates that the histone-forming metabolites within the cells can be converted to each other, beta-hydroxybutyrate could be provided from intrinsic or extrinsic sources. 

The present study showed that neither K-Ac nor K-Bu of histones were changed after long-term restriction of glucose or even after enrichment with bHB in MDA-MB231 cell line as a model of triple-negative stem-like breast cancer. Such effects of long-term treatments could result of metabolic plasticity of cancer cells (Katada et al., 2012; Martinez-Outschoorn et al., 2012a; Pavlova and Thompson, 2016; Peiris-Pagès et al., 2016; Woolf et al., 2016; Danhier et al., 2017; San-Millan and Brooks, 2017; Schwartz et al., 2017; Riester et al., 2018) which alleviated the observed induction of metabolic reprogramming by bHB and neutralized the related changes in k-But at histone proteins. Further studies are required to elucidate the exact mechanism of such plasticity and its functional impacts on the expression of different genes, 
